# Development of innate immune memory by non-immune cells during *Staphylococcus aureus* infection depends on reactive oxygen species

**DOI:** 10.3389/fimmu.2023.1138539

**Published:** 2023-05-31

**Authors:** Emmanuel Chaumond, Sandrine Peron, Nathalie Daniel, Yann Le Gouar, Éric Guédon, David L. Williams, Yves Le Loir, Gwénaël Jan, Nadia Berkova

**Affiliations:** ^1^ l'Institut national de recherche pour l'agriculture, l'alimentation et l'environnement (INRAE), Institut Agro, Science et Technologie du Lait et de l'Oeuf (STLO), Rennes, France; ^2^ Department of Surgery and Center of Excellence in Inflammation, Infectious Disease and Immunity, Quillen College of Medicine, East Tennessee State University, Johnson, TN, United States

**Keywords:** *Staphylococcus aureus*, *Lactococcus lactis*, innate immune memory, non-immune cells, epigenetics, immune response, reactive oxygen species

## Abstract

**Introduction:**

The mechanisms underlying innate immune memory (trained immunity) comprise epigenetic reprogramming of transcriptional pathways associated with alterations of intracellular metabolism. While the mechanisms of innate immune memory carried out by immune cells are well characterized, such processes in non-immune cells, are poorly understood. The opportunistic pathogen, *Staphylococcus aureus*, is responsible for a multitude of human diseases, including pneumonia, endocarditis and osteomyelitis, as well as animal infections, including chronic cattle mastitis that are extremely difficult to treat. An induction of innate immune memory may be considered as a therapeutic alternative to fight *S. aureus* infection.

**Methods:**

In the current work, we demonstrated the development of innate immune memory in non-immune cells during S. aureus infection employing a combination of techniques including Enzyme-linked immunosorbent assay (ELISA), microscopic analysis, and cytometry.

**Results:**

We observed that training of human osteoblast-like MG-63 cells and lung epithelial A549 cells with β-glucan increased IL-6 and IL-8 production upon a stimulation with *S. aureus*, concomitant with histones modifications. IL-6 and IL-8 production was positively correlated with an acetylation of histone 3 at lysine 27 (H3K27), thus suggesting epigenetic reprogramming in these cells. An addition of the ROS scavenger N-Acetylcysteine, NAC, prior to β-glucan pretreatment followed by an exposure to *S. aureus*, resulted in decreased IL-6 and IL-8 production, thereby supporting the involvement of ROS in the induction of innate immune memory. Exposure of cells to *Lactococcus lactis* resulted in increased IL-6 and IL-8 production by MG-63 and A549 cells upon a stimulation with S. aureus that was correlated with H3K27 acetylation, suggesting the ability of this beneficial bacterium to induce innate immune memory.

**Discussion:**

This work improves our understanding of innate immune memory in non-immune cells in the context of *S. aureus* infection. In addition to known inducers, probiotics may represent good candidates for the induction of innate immune memory. Our findings may help the development of alternative therapeutic approaches for the prevention of *S. aureus* infection.

## Introduction

Host immune responses are classically divided into innate and adaptive immune responses. However, it is now evident that innate immune responses demonstrate adaptive characteristics, that are termed innate immune memory or trained immunity (1). Innate immune memory is characterized by the capacity of the host to develop an intensified long-lasting (from one week to several months) immune response to subsequent unrelated challenges independent of the adaptive immunity (2). The molecular mechanisms underlying innate immune memory include metabolic and epigenetic alterations, such as DNA methylation and histone acetylation, leading to the functional reprogramming of cells (3). It was originally established that innate immune memory could be developed by immune cells such as monocytes, macrophages, and natural killer cells, which underwent a phenotypic reprogramming following exposure to stimuli that influence cell reaction to infections (4). However, the lifespan of immune cells is shorter than the duration of innate immune memory. Consequently, the development of innate immune memory by non-immune cells was suggested (5). Indeed, innate immune memory was described in hematopoietic and mesenchymal stem cells or fibroblasts (5). Different active compounds, such as heat-killed *Candida albicans*, (1→3)β-glucan (β-glucan), a fungal cell wall compound, zymosan, or muramyl dipeptide (MDP) component of peptidoglycans, induce innate immune memory (6). Microbial ligands β-glucan and MDP induce innate immune memory in host cells through dectin-1 and nucleotide-binding oligomerization domain 2 (NOD2) respectively (7, 8).

It was shown that the development of innate immune memory affords a wide-ranging advantage during lethal bacterial infections, including *Staphylococcus aureus* infections (2, 9). This opportunistic pathogen, is responsible for a multitude of diseases ranging from mild skin infections to life-threatening disseminated infections such as pneumonia, endocarditis, meningitis and osteomyelitis that are prone to recurrence (10). *S. aureus* small colony variants are frequently associated with chronic infection (11). Treatment of devastating osteomyelitis infections is challenging because of the poor bioavailability of antibiotics in bone tissue, of antibiotic resistance, and of the biofilm-like properties of the infection (12). Staphylococcal pneumonia can lead to complications such as severe necrotizing pneumonia, bacteremia, or sepsis with shock (13). *S. aureus* also constitutes a serious problem in veterinary medicine, e.g. cattle chronic mastitis caused by *S. aureus* bacteria is extremely difficult to treat (14). The protective role of innate immune memory was established in a mouse model of systemic staphylococcal infection (9), as well as in the protection against recurrent methicillin-resistant *S. aureus* (MRSA) skin and skin structure infection (SSSI) (15). It relies on specific memory conferred by macrophages. However, the role of non-immune cells in the development of innate immune memory is not completely elucidated. We were interested in the investigation of the induction of innate immune memory towards *S. aureus* infection in osteoblast-like MG-63 cells that are used as a model of osteomyelitis. To examine whether this prospective phenomenon is a general trait of non-immune cells, lung epithelial A549 cells that are used as a model of the respiratory tract epithelium interacting with external environment including microorganisms, were selected as another cell line for this study.

An involvement of IL-6 and IL8 in the host response was established in the context of *S. aureus* infection. Indeed, transcriptional profiling of MG-63 cells containing internalized *S. aureus* demonstrated a high expression of IL-6 and IL-8 (16). *S. aureus* triggers a production of IL-1*β*, TNF, and IL-6 in a fresh human whole blood and a release of IL-8 in purified polymorphonuclear neutrophils (17). An assessment of production of proinflammatory cytokines such as IL-6 and IL-8 is widely used as a readout for the characterization of an immune innate memory against various pathogens including *S. aureus* (18). Development of innate immune memory relies on epigenetic regulation of gene expression that is determined by chromatin reorganization (19). Recently it was demonstrated that histone H3 acetylation on lysine residues leads to the opening of chromatin structure (20). This process increases the accessibility of the DNA to the transcriptional machinery and regulatory elements, promoting and facilitating enhanced transcription (21).

An assessment of histone H3 acetylation at lysine 27 (H3K27), which marks active promoters and distal regulatory elements (enhancers), in human primary monocytes identified a large number of promoters that were specifically induced by β-glucan training (22). In addition, the authors showed an increased IL-6 production by β-glucan trained human primary monocytes after stimulation with *S. aureus* (22). Moreover, an involvement of H3K27 acetylation was demonstrated in β-glucan-trained macrophages (23). Therefore, an assessment of H3K27 acetylation together with IL-6 and IL-8 production was used as functional readouts for an analysis of innate immune memory against *S. aureus* by non-immune osteoblast-like MG-63 and lung epithelial A549 cells in the current investigation.

Induction of innate immune memory may be considered as an additional therapeutic alternative to fight *S. aureus* infection. Likewise known inducers, probiotics, which confer a benefit to the host health, may represent good candidates for the induction of innate immune memory (24, 25)

In the current work we investigated the development of innate immune memory in human osteoblast-like MG-63 cells and lung epithelial A549 cells against *S. aureus*, by using β-glucan and MDP as inducers. Production of pro-inflammatory cytokines IL-6 and IL-8 and epigenetic alterations were measured as functional readouts of innate immune memory. To verify whether a histone acetylation resulting in enhanced gene transcription of proinflammatory cytokines was mediated by oxidative stress, we evaluated a potential correlation between H3K27 acetylation and IL-6 and IL-8 production by using N-Acetylcysteine (NAC), a known inhibitor of reactive oxygen species (ROS), in our model of innate immune memory against *S. aureus.*

We furthermore investigated the ability of *Lactococcus lactis*, a food-grade lactic acid bacterium with probiotic properties, to induce such innate immune memory. The current study improves our understanding of innate immune memory of non-immune cells and may lead to the development of new approaches for the prevention of *S. aureus* infection.

## Materials and methods

### Maintenance of eukaryotic cells

The human osteoblast-like MG-63 cell line (LGC Standards, Teddington, UK) is derived from the left femur of a 14-year-old male (26). MG-63 cells were cultured in cDMEM (StableCell DMEM High glucose (Sigma), 10% fetal calf serum (FCS, BioWest, Nuaille, France) supplemented with 100 U/mL penicillin, and 100 μg/mL streptomycin). Type II pneumocyte cell line A549 derived from a human lung (American Type Culture Collection) was maintained in Kaighn’s modification of HAM’s F12 medium (Gibco, Fisher Scientific Illkirch, France) supplemented with 10% of FCS (BioWest, Nuaillé, France), 100 U/mL penicillin, and 100 μg/mL streptomycin (Sigma). All incubations were at 37°C in 5% CO_2_. Trypsin/EDTA (Sigma) was used to release adherent cells for cells subculturing.

### Bacterial strains and culture conditions

#### Staphylococcus aureus

The MW2 methicillin resistant *Staphylococcus aureus* (MRSA) strain was provided by the Laboratory of Human Bacterial Pathogenesis, National Institute of Health (Bethesda, MD, USA) and was cultivated as described previously (27). Briefly, aliquots of frozen *S. aureus* bacteria were cultivated overnight in Brain Heart Infusion (BHI, Bacto TM BHI, Becton Dickinson) broth at 37°C, then 1 mL of overnight culture was diluted (1:50) in high glucose DMEM medium (Dominique Dutscher, Bernolsheim, France). Strains were grown in 50 mL tubes at 37°C under agitation until cultures had reached an optical density of 0.6 at 600 nm, corresponding to approximately 10^8^ colony-forming unit per milliliter (CFU/mL). Bacteria were harvested by centrifugation and resuspended in the interaction medium (DMEM). Bacterial concentrations were estimated spectrophotometrically and the number of live bacterial cells was confirmed by plate counts on BHI agar.

#### Lactococcus lactis

The *Lactococcus lactis* subsp. *cremoris* reference strain MG1363 was provided by CIRM-BIA (Centre International de Ressources Microbiologique, Bactéries d’Intérêt Alimentaire, Rennes, France). *L. lactis* was cultivated in M17 medium (BD biosciences, Le Pont de Claix Cedex, France) supplemented with 0.5% of glucose (M17G), in 50 mL tubes, at 30°C, without agitation, as described (28). Cultures were harvested in final stationary phase, where optical density at 600 nm was 2.5, corresponding to a population of 1.5 x 10^9^ CFU/mL. Bacteria were centrifuged (10 000 x g, 4°C, 10 min) and resuspended in PBS containing 20% glycerol, in calibrated bacterial suspensions containing 10^9^ CFU/mL of lactococci, prior to storage at -80°C. This population was checked by CFU counting on M17G medium added with agar. Upon thawing, lactococci suspensions were diluted in the interaction medium (DMEM).

### Glucan extraction and inducers storage

Glucan was isolated from the yeast phase of *C. albicans* SC5314 using the method previously described by our laboratory (29, 30). β-1,3-(D)-Glucan was produced in the laboratory of David L. Williams (TN, USA). Briefly, lyophilized *C. albicans* was extracted with 0.75 N NaOH (3x) and 0.5 N H3PO4 (2x) at 100°C for 15 min, followed by extraction with absolute ethanol (90°C) to remove lipids. The resulting product was a water insoluble particulate (~3µm) that was harvested and washed by centrifugation. The glucan was washed (3x) in dH2O, depyrogenated, sterilized, suspended in dextrose 5% (w/v) and water and stored at 4°C until used. The identity and structure of the glucan was confirmed by NMR (30). The product was >95% (1→3,1→6)- β-D-glucan. Sterility and endotoxin testing of the glucan was performed as described: there was no microbial contamination and no detectable endotoxin (30). Aliquots of (1→3,1→6)-β-D-glucan were prepared in sterile conditions and stored at 4°C.

Muramyl dipeptide (MDP) was obtained from InvivoGen Europe (Toulouse, France). MDP was dissolved in sterile water, aliquoted under sterile conditions, and stored at a temperature of -20°C.

### Development of innate immune memory model in human non-immune cells

The *in vitro* model used to study innate immune memory in MG-63 and A549 cells was developed according to the method described for human primary monocytes (31).

**Figure 1:**
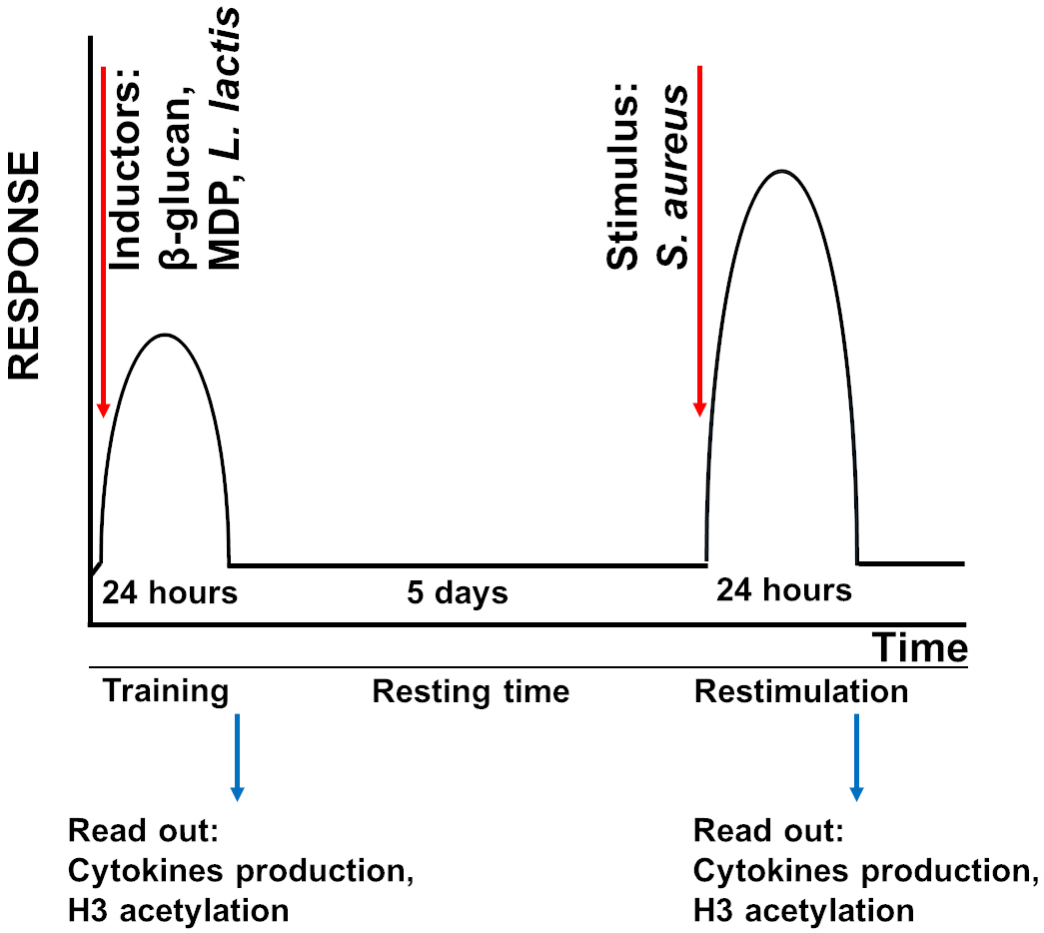
Schematic overview of the innate immune memory method. Either MG-63 or A549 cells were trained with inducers of innate immune memory (β-glucan, MDP) or *L. lactis* for 24 h. After the training, inducers were washed out and cells were rested for 5 days. Cells were then restimulated with *S. aureus* for 24 h.

Either MG-63 or A549 cells, both being adherent cell line, (20,000 cells/well) were added to flat-bottom of 24-well cell culture plates and were grown in the corresponding medium for 24 h at 37°C. After washing with PBS, cells were treated with different inducers of innate immune memory (β-glucan or MDP) (trained cells) or were cultivated in the medium (non-trained and control cells) for an additional 24 h (the training phase) (**Figure 1**). Different concentrations of inducers (10 µg/mL and 50 µg/mL of MDP; 50 µg/mL and 100 µg/mL of β-glucan) were evaluated in order to develop the optimal *in vitro* model. Afterwards, the control cells, cells treated with inducers (trained cells) or cells exposed to a cultivation medium (non-trained cells) were washed with PBS and incubated for 5 days (the resting time) in corresponding culture medium, and the medium was changed once at day 3 (**Figure 1**). In the preliminary experiments, viability and cell concentration were assessed for each condition at every time point. Cells viability was measured using the colorimetric MTT [3-(4,5-dimethylthiazol-2-yl)2,5-diphenyltetrazolium bromide] assay Tox-1 kit (Sigma, Saint-Quentin Fallavier, France) according to the manufacturer’s recommendations and as we described previously (32). Absorbance was measured at 570 nm with a reference filter of 630 nm using a plate reader (Molecular Devices, Gregoire, France). There was no difference in the absorbance of cells exposed to 10 µg/mL (MG-63 cells) and 50 µg/mL of MDP or to 50 µg/mL and 100 µg/mL of β-glucan (MG-63 and A549 cells) compared to control cells at the same time point. Furthermore, in additional samples, cell concentrations were determined using a Cell counter (Bio-Rad, Marnes-la-Coquette, France). The results obtained using cell counting corresponded to those obtained using the MTT assay kit. There was no difference in the concentration of cells exposed to 10 µg/mL and 50 µg/m of MDP or to 50 µg/mL and 100 µg/mL of β-glucan compared to control cells at the same time points. Altogether, the results indicate that the inducers being studied do not have a harmful effect on the host cells. These observations are in line with the previously demonstrated absence of alteration in proliferation rate of MG-63 cells, when treated with β-glucan at concentrations lower than 300 µg/mL (33).

Cells were then infected with *S. aureus* with a multiplicity of infections (MOI, number of bacteria per cell at the onset of infection) of 60:1 and 100:1. Cell concentrations at the time bacteria were added were determined using one of the four samples prepared for each MOI. The remaining samples were used for the analysis in triplicate.

Non-internalized bacteria were removed 100 min after infection by washing the wells with PBS, followed by incubation in cDMEM with 3% FCS containing 100 mg/mL gentamicin and 20 mg/mL of lysostaphin for 2 h, which eliminates the extracellular and cell-adherent *S. aureus* from eukaryotic cell-bacterial mixtures *in vitro* (34) followed by incubation in cDMEM containing 25 mg/mL of gentamicin for 24 h (restimulation time). The corresponding medium was replaced in the wells containing trained, non-trained and control cells at the same time to ensure that the culture conditions were the same.

To determine if *S. aureus* exhibits cytotoxicity on eukaryotic cells, we performed preliminary experiments using both the MTT test and cell counting via Cell counter. Our results showed no significant difference in the absorbance or number of MG-63 cells exposed to *S. aureus* (MOI 60:1 or 100:1), as described above, compared to control cells. Thus, our findings suggest that *S. aureus* does not display cytotoxicity towards MG-63 cells in the described experimental setup.

The experiments conducted with A549 cells exposed to *S. aureus* at an MOI of 60:1 yielded similar results, as there was no significant difference in either the absorbance as determined by the MTT test, or the number of A540 cells when compared to control cells. However, when the MOI was increased to 100:1, a decrease in the absorbance (the MTT test) was observed, which was consistent with a 15% decrease in the cell count compared to uninfected cells. These findings suggest that increasing the MOI of *S. aureus* exposure can lead to a reduction in cell viability, suggesting that an exposure of A549 cells to *S. aureus* at the MOI 100:1 has a relatively weak cytotoxic effect on A549 cells. However, there was no difference between the number of viable trained and non-trained A549 cells exposed to *S. aureus* with the MOI 100:1 that allowed us to evaluate the production of interleukins as a readout of innate immunity in both MG-63 and A549 cell lines exposed to *S. aureus* at MOIs of 60:1 and 100:1.

In subsequent experiments, the number of remaining A549 or MG-63 cells was quantified using a cell counter following supernatant collection at each time point for all conditions.

Bacterial concentrations were estimated spectrophotometrically and were confirmed by plate counting on the appropriate solid medium.

Cell supernatants were collected by a centrifugation, the number of remaining adherent cells were determined using Cell counter and compared to the number of control untreated cells that allows to assess the cell concentration and the viability in every well for each condition at each time point. The number of viable cells was increased from 20,000 at the beginning experiment to ~ 300,000 cells by the end of experiment. The findings suggest that only a portion of the cells were primed with inducers at the beginning of the experiment, rather than the entire population being primed at the end.

Supernatants were obtained from various experimental conditions, (i) control untreated cells, (ii) cells treated with an inducer for 24 h, followed by a resting period of 5 days and an incubation for additional 24 h (iii) cells incubated in a cultivation medium for 24 h, followed by a resting period of 5 days before being exposed to *S. aureus*, and (iv) cells trained with an inducer for 24 h, followed by a resting period, before being exposed to *S. aureus*. Supernatants were stored at -20°C until cytokine measurement.

### Quantification of IL-6 and IL-8 production by MG-63 and A549 cells using ELISA

Cell culture supernatants (undiluted and at dilutions 1:10 and 1:100) were subjected to human IL-6 detection kit (Thermofischer Life Technologies, INVITROGEN) by ELISA according to the manufacturer’s instructions using a suggested dilution of provided solutions. Briefly, wells of 96-well plates were coated with capture anti-human IL-6 or antibody and were incubated overnight at 4°C. Each step of ELISA was followed by washing with the washing solution. After the saturation of binding site with a blocking solution for 2 h, the tested samples were then added to the appropriate wells. A standard curve was done using twofold serial dilutions of a standard solution containing 200 pg/mL of IL-6. Recombinant human IL-6 provided in the kit were used for generating the standard curve and calibrating samples. After an overnight incubation, a detection biotin-conjugated anti-human IL-6 antibody was added to the wells for 1 h, followed by the incubation with the streptavidin-horseradish peroxidase conjugate solution for 30 min. The substrate solution of tetramethylbenzidine was then added to the wells for 30 min in the dark. After an addition of a stop solution (the phosphoric acid, Sigma), the absorbance was read at 450 nm with the BIOTEK 800TS micro plate reader (BioTech, Lyon, France).

For the detection of human IL-8 a sandwich-ELISA kit (BD Bioscience, Le Pont de Claix, France) was used according to the manufacturer’s instruction. Briefly, a monoclonal antibody specific for IL-8 coated on a 96-well plate and were incubated overnight at 4°C. Cell culture supernatants (undiluted and at dilutions 1:10, 1:200, 1:800) or standard recombinant IL-8 or were added to the wells. A standard curve was done using twofold serial dilutions of a standard solution containing 200 pg/mL of IL-8. After washing with the washing solution, a streptavidin-horseradish peroxidase conjugate mixed with biotinylated anti-human IL-8 antibody was added to the wells. The wells were washed and tetramethylbenzidine substrate solution then was added for 15 min, followed by the addition of a stop solution (phosphoric acid) for 15 min. Microwell absorbances were read at 450 nm with the BIOTEK 800TS micro plate reader (BioTech, Lyon, France).

The sensitivity of either IL-6 or IL-8 detection is 2 pg/mL. The concentration of interleukins was calculated according to the standard curve. After collecting supernatants corresponding to each experimental condition and time point, cytokine production was normalized to the number of adherent cells in each well, as described above. At least 3 replicates were performed in each experiment. Coefficient of variation for ELISA was approximately 10%.

### Estimation of histone modifications by confocal microscopy

Either A549 or MG-63 cells were seeded in 8-wells Labtek culture chamber (Nunc) for 24 h. They were then treated for the development of innate immune memory in non-immune cells during *S. aureus* infection as described above. Briefly, cells were treated with 100 µg of β-glucan for 24 h (the training time), followed by the 5 days resting time. Cells were then exposed to *S. aureus* (the restimulation time) for 100 min, afterwards treated with lysostaphin and gentamicin as described above. Following a 24-hour incubation period, the supernatants of the cells were assessed for IL-6 and IL-8 production using ELISA, while the remaining cells were analyzed for histone acetylation markers. The remaining cells were fixed with PFA 4% in PBS for 20 min at room temperature, permeabilized with Triton X100 0.3% for 30 min, treated with 2% BSA for 100 min, and incubated overnight at 4 °C with a 1:500 dilution of primary antibody H3K27ac (#39134, Active Motif, Carlsbad, CA, USA) in 2% BSA. This antibody recognises histone H3 acetyl-lysine 27. Afterwards cells were incubated with the secondary antibody (# A32733, Goat anti-Rabbit IgG, Highly Cross-Adsorbed Secondary Antibody Alexa Fluor™ Plus 647 Thermofisher scientific, Illkirch-Graffenstaden, France, diluted 1:1000) for 100 min, and were finally mounted under coverslide with VECTASHIELD DAPI PLUS (Eurobio Scientific, Les Ulis, France). To exclude an unspecific binding in preliminary experiments we used an isotype control rabbit IgG antibody (Sigma-Aldrich) instead of primary antibody in the same conditions. Control isotype antibody was of the same class and immunoglobulin type as primary antibody H3K27ac, but has no specificity for any known epitope (# NI01, Normal rabbit IgG, Sigma-Aldrich). In some experiments, only a secondary antibody was applied. There was no staining neither when an isotype control antibody nor only a secondary antibody was used (**Supplementary Figure 1**).

Confocal laser scanning microscopy (CLSM) observation was performed using a ZEISS LSM 880 inverted confocal microscope (Carl Zeiss AG, Oberkochen, Germany) equipped with immersion objective 63× (Plan Apochromat objective, oil immersion, NA 1.4) driven by Zen software. The pinhole is set to the one Airy unit. The images were captured with strictly identical acquisition settings for every sample. Secondary antibody coupled with Alexa Fluor™ and DAPI were detected using a GaasP between 540 and 735 nm and a PMT between 395 and 530 nm, respectively. The experiments were conducted in a blinded manner, and for the quantitative analysis, five randomly selected fields were used from each sample.

In order to compare posttranslational modifications of histones in different samples, we employed the semi-quantitative method for an evaluation of the fluorescence intensity as described previously (35) with some modifications.

The Integrated Density (ID) of the selected field of the image from the confocal microscope in the red channel corresponding to post-translational modifications of histones was measured using the ImageJ (FIJI) software. After subtraction of the density of the background, ID was divided by the cell number in the selected field to obtain the normalized ID.

To obtain a 3D images of the analyzed samples, multiple confocal images acquired at different “z” focal plans from the bottom to the top of the slide culture chamber in the labeled preparation. Reconstituted 3D images of *S. aureus*-infected host cells were obtained by Z-stack images using a Zeiss ZEN software.

### Estimation of histone modifications by flow cytometry

For an estimation of histone acetylation by flow cytometry, we used the method described previously (36, 37) with some modifications. Cells were treated with an inducer of innate immune memory as indicated above. Briefly, MG-63 cells (20,000 cells/well) were cultivated in 24-well cell culture plates for 24 h. After an exposure to 100 µg/mL of β-glucan (training) or medium for 24 h, the cells were rested for 5 days. Cells (trained or non-trained) were exposed to *S. aureus* (MOI 60:1) for 100 min. Non-internalized bacteria were eliminated by incubating cells in cDMEM with gentamicin (100 mg/mL) and lysostaphin (20 mg/mL) for 2 h, followed by gentamicin (25 mg/mL) in cDMEM for 24 h (restimulation). As a positive control for histone acetylation, an incubation of cells for 24 h with 10 nM of Trichostatin A (TSA), known inhibitor of the enzymatic activity of histone deacetylases (HDACs) (38) was used. A binding of TSA to HDCA results in the inhibition of the elimination of acetyl-group from HDCA substrates leading to a strong increase of acetylated proteins in the cell, from which a majority is composed of histones (36).

After incubation, adherent cells were dissociated into single-cell suspension using Trypsin/EDTA treatment. They were then fixed in 4% paraformaldehyde/PBS for 20 min at room temperature and permeabilized in 0.3% TritonX100/PBS for 10 min. Cells were subsequently incubated with anti-H3K27ac antibody (# 15562, Acetyl-Histone H3 (Lys27) (D5E4) XP^®^ Rabbit mAb, PE Conjugate, Cell Signaling Technology Europe, Leiden, Netherlands) diluted in PBS/2%BSA (1:50) for 1 h. An isotype control antibody, which is not directed against any known antigen (#5742, Rabbit (DA1E) mAb IgG XP^®^ Isotype Control, PE Conjugate, Cell Signaling Technology Europe, Leiden, Netherlands) was used to exclude nonspecific binding. After 3 rinses, cells were resuspended in PBS and were analyzed for H3K27 acetylation using an Accuri C6 flow cytometer. The instrument is equipped with a filter for PE fluorescence detection (FL2 channel). Data were collected from 20,000 cells and analyzed using the CFlow software (Becton Dickinson, Le Pont de Claix, France) as described (37). Cells were analyzed using FSC-A x SSC-A plot. The major density of events was captured by the gate. The events corresponding to debris, cell fragments, and pyknotic cells were eliminated. Values of the respective mean fluorescence intensities (MFIs) were compared to that of the control.

### Analysis of the involvement of ROS in the development of innate immune memory by non-immune cells

Osteoblast-like MG-63 or lung epithelial A549 cells (20,000 cells/well) were seeded in wells of flat-bottom 24-well plates. When indicated cells were pretreated with 1 mM of the ROS scavenger N-Acetylcysteine (NAC) for 1 h prior to β-glucan exposure for 24 h or pretreated with NAC for 24 h, followed by the incubation for 5 days before an infection with *S. aureus* at MOIs 60:1. Unbound bacteria were removed by the incubation with 20 mg/mL of lysostaphin and 100 mg/mL gentamicin for 2h. Determination of the cell concentration (according to the method described above) in wells containing untreated control cells and those exposed to 1 mM NAC did not reveal any significant difference showing that the addition of 1 mM NAC did not have a significant effect on cell proliferation rates, which is consistent with a previous research (39).

Cell supernatants were collected (i) from control untreated cells at the end of experiment, (ii) from cells incubated with a cultivation medium for 24 h, followed by a 5 day-resting period before being exposed to *S. aureus* for 24 h, (iii) from cells pretreated with NAC for 24 h, followed by a 5 day-resting period before *S. aureus* infection, (iv) from cells pretreated with β-glucan for 24 h, followed by a 5 day-resting period before *S. aureus* infection, (v) from NAC-treated cells for 1 h before an exposure to β-glucan and NAC for 24 h, followed by a 5 day-resting period before being exposed to *S. aureus*. The production of IL-6 and IL-8 was measured by ELISA in cell supernatants. IL-6 and IL-8 concentrations were normalized by the number of cells in the aliquots of cells from each well that were collected after a trypsin/EDTA treatment. The remaining cells were used for the analysis of an H3K27 acetylation by a flow cytometry as described above in order to determine if there is a correlation between interleukins production and histone acetylation. An employment of NAC, a known ROS inhibitor, further determined whether a histone acetylation resulting in the enhanced gene transcription of proinflammatory cytokines is mediated by oxidative stress.

### Evaluation of the capacity of *Lactococcus lactis* to induce innate immune memory in human non-immune cells

To evaluate the potential cytotoxicity of *L. lactis* on A549 and MG-63 cells, we conducted preliminary experiments in which 20,000 cells were seeded per well and allowed to incubate for 24 h, followed by the exposure to *L. lactis* at MOIs 100:1, 500:1 and 1000:1 for 100 minutes. Non-internalized *L. lactis* were removed by incubating the cells with gentamicin (100 mg/mL for 2 hours followed by 25 mg/mL for 24 h), followed by a 5 day-resting time and additional 24 h of incubation. Cell viability was assessed using the MTT assay, as described previously. Additionally, cell counts were obtained using a cell counter. Absorbance values from the MTT assay and cell counts of cultures exposed to different concentrations of *L. lactis* were compared to untreated control cells at the same time point: 24 h after *L. lactis* addition and at the end of experiment. Our results showed no significant differences in the absorbance or number of A549 or MG-63 cells exposed to *L. lactis* at an MOI of 100:1 compared to untreated control cells. These findings suggest that exposure to *L. lactis* at an MOI of 100:1 did not result in any detectable cytotoxic effects on host cells. Based on these results, an MOI of 100:1 was selected for the evaluation of the capacity of *L. lactis* to induce the innate immune memory in MG-63 and A549 cells. Experiments were performed using the same method as described above for the induction of the innate immune memory by β-glucan or MDP, except that *L. lactis* was used as the stimulus. Briefly, MG-63 or A539 cells were either exposed to medium for 24 h or to *L. lactis* (MOI 100:1) for 100 minutes. Non-internalized *L. lactis* were removed by incubation in cDMEM with gentamicin (100 mg/mL) for 2 hours, followed by 25 mg/mL for 24 h as a training period. After a resting period of 5 days in cDMEM with 25 mg/mL gentamicin, the cells were infected with *S. aureus* in cDMEM (MOI 60:1). Unbound *S. aureus* bacteria were removed 100 min after infection, using lysostaphin and gentamicin as described above. Supernatants were collected (i) from control untreated cells at the end of the experiment, (ii) from cells exposed to *L. lactis*, followed by a 5-day resting time and an incubation for additional 24 h, (iii) from cells exposed to the cultivation medium for 24 h, followed by a 5-day resting time before being exposed to *S. aureus*, and (iv) from cells exposed to *L. lactis* followed by a 5-day resting time before being exposed to *S. aureus*. Supernatants were stored at -20°C until cytokine measurement.

In order to investigate the potential correlation between epigenetic alterations and cytokine production, the acetylation of H3K27 was evaluated in the same experiment. After collecting supernatants for determining IL-6 and IL-8 production, the remaining cells were analyzed for H3K27 acetylation using flow cytometry as described above.

### Statistical analysis

All experiments were conducted at least in triplicate and repeated 3 times. The values are expressed as mean ± standard deviation ( ± SD). The differences among the groups corresponding to different treatments were assessed by analysis of variance (ANOVA) followed by a Tukey’s Honestly Significant Difference test in R software. (*) P-values **≤** 0.05 were considered to be significant.

## Results

### Setting up the initial conditions for the model of innate immune memory in non-immune human osteoblast-like MG-63 cells

Firstly, the initial conditions of experiments were determined. Since cells cease to proliferate at a high density and enter a quiescence state (32, 40), we tested different amounts of FCS and various MG-63 cell concentrations at the beginning of the experiment to ensure cell proliferation during the entire experiment (7 days).

**Figure 2:**
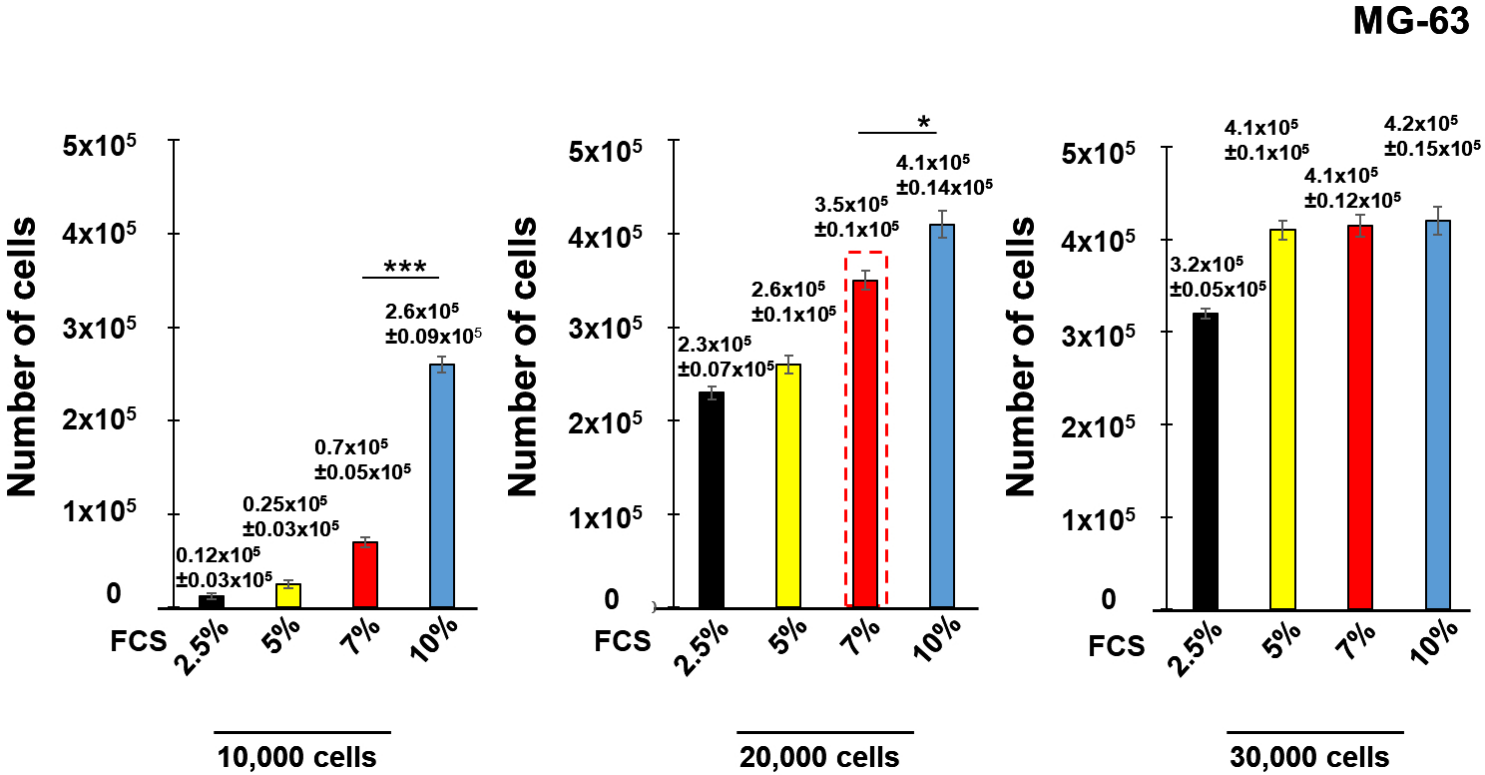
Development of the model of innate immune memory in non-immune human osteoblast-like MG-63. Human osteoblast-like MG-63 cells were seeded with an initial concentration of 10,000 cells/well, 20,000 cell/well or 30,000 cell/well into 24 well-plates in the medium containing either 2.5%, 5%, 7% or 10% of FCS. The number of cells at the end of the experiment (7^th^ day) was determined by a Cell counter (Bio-Rad) using samples prepared for each concentration of FCS corresponding to the different initial cell concentrations. Data are presented as mean +/- SD. Each experiment was done in triplicate. The differences among the groups corresponding to different conditions were assessed by analysis of variance (ANOVA) followed by a Tukey’s Honestly Significant Difference test. (*) P-values, ≤ 0.05 and (***) P-values ≤ 0.005 were considered to be significant.

Seeding with an initial cell concentration of 10,000 cell/well in the medium containing 10% of FCS did not reach confluence at the end of the experiment. Lower serum concentrations (2.5%, 5% and 7% of FCS) did not allow cell growth (**Figure 2**). Seeding with an initial concentration of 20,000 cells/well and 30,000 cells/well containing 10% of FCS for both initial cell concentrations resulted in cell confluence of the cell culture (~400,000 cells), as well as for the cell culture with 30,000 cells/well containing 5% and 7% of FCS. Cells reached 90% of confluence with an initial concentration of 20,000 cells/well containing 7% of FCS (~300,000 cells). Consequently, an initial concentration of 20,000 cell/well containing 7% of FCS (red dashed line) was selected for further experiments. The same conditions were employed in the experiments with human lung epithelial A549 cells.

### Training of osteoblasts-like MG-63 cells with MDP or β-glucan increases subsequent production of pro-inflammatory cytokines IL-6 and IL-8 upon a stimulation by *S. aureus*

The assessment of the development of innate immune memory against *S. aureus* in MG-63 cells was performed using the method described in the Materials and Methods section (**Figures 1**). Using ELISA we monitored the production of pro-inflammatory cytokines IL-6 and IL-8 in non-immune osteoblast like MG-63 cells exposed to *S. aureus* following stimulation by two known inducers of innate immune memory, β-glucan and MDP, in order to check whether these cells can develop an innate immune memory against *S. aureus*.

**Figure 3:**
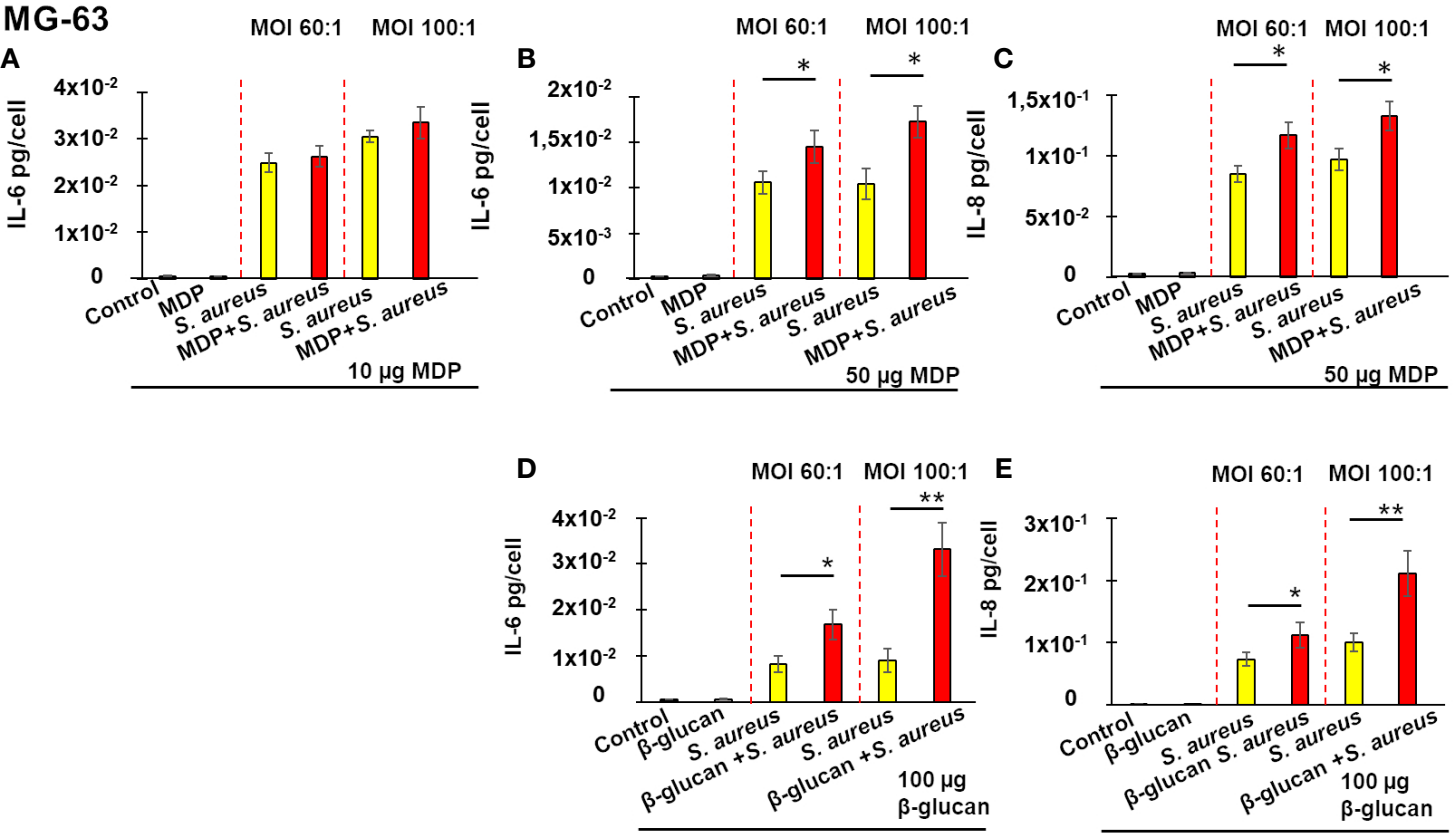
Training of osteoblasts-like MG-63 cells increases a subsequent production of IL-6 **(A, B, D)** and IL-8 **(C, E)** upon a stimulation by *S. aureus*. Osteoblast-like MG-63 cells (20,000 cells/well) were seeded in wells of flat-bottom 24-well plates. Afterwards, the cells were either pre-treated with β-glucan or MDP, or cultivated in the growth medium for 24 h, followed by a 5-day incubation prior to infection with *S. aureus* for 100 minutes, and then incubated for an additional 24 h as described in Material and Methods. Supernatants were collected (i) from control cells at the end of experiment (black bar graph), (ii) from cells exposed solely to inducers followed by a 5-day resting time and then incubated for an additional 24 h (grey bar graph), (iii) from cells incubated in the cultivation medium for 24 h, followed by a 5-day resting time before an exposure to *S. aureus* (yellow bar graph) and (iv) from inducer pretreated-cells that were infected with *S. aureus* after a 5-day resting time (red bar graph). The production of IL-6 and IL-8 was measured by ELISA. Data are presented as mean +/- SD. The vertical red dashed lines differentiate bar graphs corresponding to MOI’s 60:1 and 100:1. Each experiment was done in triplicate. The differences among the groups corresponding to different treatments were assessed by analysis of variance (ANOVA) followed by a Tukey’s Honestly Significant Difference test. (*) P-values ≤ 0.05 and (**) P-values ≤0.01 were considered to be significant.

As is shown on **Figure 3**, exposure of MG-63 cells to either 10 µg or 50 µg of MDP for 24 h, followed by a 5 days-incubation, did not modify production of IL-6 or IL-8, when compared to control untreated cells. Cultivation of cells in the cultivation medium for 24 h followed by a 5 day-resting time before an exposure to *S. aureus* for 100 min and incubation for 24 h resulted in increased IL-6 production (**Figure 3A**, yellow bar). Pre-treatment of MG-63 cells with 10 µg/mL of MDP, followed by a 5-days resting time, did not alter IL-6 production upon a stimulation by *S. aureus*, whether the MOI was 60:1 or 100:1 (**Figure 3A**, red bar).

By contrast, pre-treatment of MG-63 cells with 50 µg/mL of MDP, followed by a 5-days resting time, resulted in a significant increase of IL-6 production upon a stimulation with *S. aureus* (red bar), compared to cells exposed to a cultivation medium, followed by a 5 day-resting time before an infection with *S. aureus* (yellow bar), whether the MOI was 60:1 or 100:1 (**Figure 3B**). A similar increase in IL-8 production upon a stimulation by *S. aureus* was observed after MG-63 cells were pretreated with 50 µg/mL of MDP, followed by a 5-days resting time, compared to cells exposed to the cultivation medium, followed by a 5 day-resting time before an exposure to *S. aureus* at MOI either 60:1, or 100:1 (**Figure 3C**).

There was no difference in an interleukins production between control untreated cells and cells exposed to 100 µg of β-glucan (a known inducer of innate immune memory) for 24 h, followed for a 5 days-incubation. The training of MG-63 cells with 100 µg β-glucan, followed by a 5-days resting time, significantly increased the IL-8 and IL-6 production upon a stimulation by *S. aureus* (red bar), compared to non-trained cells exposed to *S. aureus* following a 5-days resting time (yellow bar) (MOI 100:1) (**Figures 3D, E**). There was no effect when lower concentrations of β-glucan were used (data not shown).

It is important to note that the cell counting analysis did not reveal any significant difference in the number of trained and non-trained MG-63 cells that were exposed to *S. aureus* at MOI 60:1 or 100:1.

### Training of lung epithelial A549 cells using MDP or β-glucan triggers enhanced production of IL-6 and IL-8 upon a stimulation by *S. aureus*

We asked whether the development of innate immune memory in non-immune osteoblast-like MG-63 cells towards *S. aureus* was restricted to organ-specific cell line, or was a general phenomenon. In this aim, we investigated the ability of another non-immune cell line, human lung epithelial A549 cells, to develop an innate immune memory. The same conditions were used for both cell lines.

**Figure 4:**
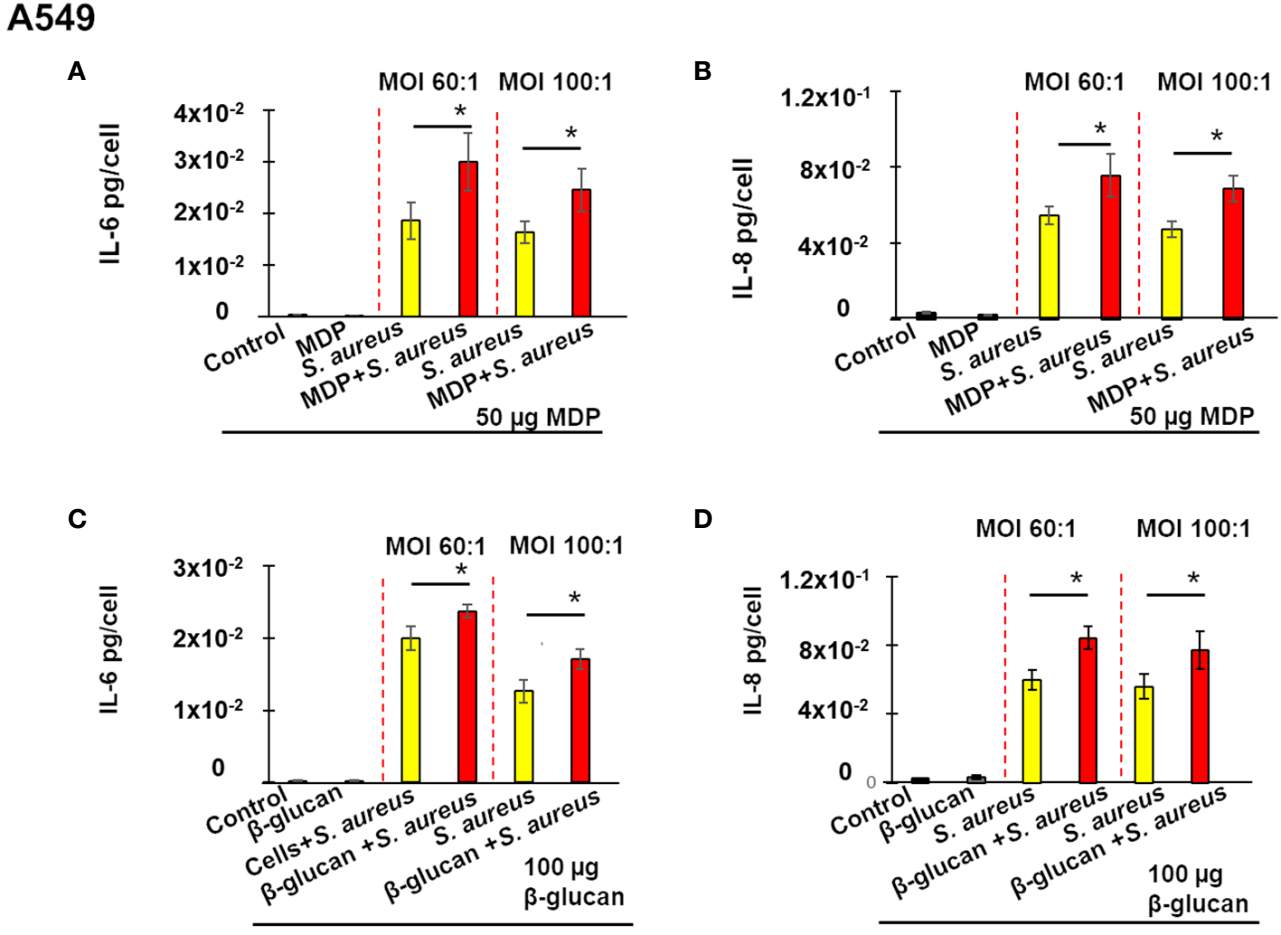
Training of lung epithelial A549 cells triggers an enhanced production of IL-6 **(A, C)** and IL-8 **(B, D)** upon a stimulation by *S. aureus*. Human lung epithelial A549 cells (20,000 cells/well) were seeded in wells of flat-bottom 24-well plates. Afterwards, the cells were either pre-treated with β-glucan or MDP, or cultivated in the growth medium for 24 h, followed by a 5-day incubation prior to infection with *S. aureus* for 100 minutes, and then incubated for an additional 24 h as described in Material and Methods. Supernatants were collected (i) from control cells at the end of experiment (black bar graph), (ii) from cells exposed solely to inducers followed by a 5-day resting time and then incubated for an additional 24 h (grey bar graph), (iii) from cells incubated in the cultivation medium for 24 h, followed by a 5-day resting time before an exposure to *S. aureus* (yellow bar graph) and (iv) from inducer pretreated-cells that were infected with *S. aureus* after a 5-day resting time (red bar graph). The production of IL-6 and IL-8 was measured by ELISA. The vertical red dashed lines differentiate bar graphs corresponding to MOI’s 60:1 and 100:1. Data are presented as mean +/- SD. Each experiment was done in triplicate. The differences among the groups corresponding to different treatments were assessed by analysis of variance (ANOVA) followed by a Tukey’s Honestly Significant Difference test. (*) P-values ≤ 0.05 were considered to be significant.

As is shown on **Figure 4**, neither IL-6 nor IL-8 was modulated by pretreatment with to 50 µg of MDP for 24 h, followed by a 5-days resting period. This pretreatment, followed by a 5-days resting period however induced a significant increase in IL-6 production upon a stimulation by *S. aureus* at MOI 60:1, or 100:1 (**Figure 4A**). A similar increase in IL-8 production upon *S. aureus* stimulation was observed in A549 cells pretreated with 50 µg/mL MDP. The production of IL-8 was also increased upon exposure of pretreated cells to *S. aureus* at MOI 60:1, or 100:1 (**Figure 4B**).

Similar results were obtained when A549 cells were pre-treated with 100 µg/mL of β-glucan. There was no difference, between control untreated cells and cells exposed to 100 µg of β-glucan for 24 h, after the 5-days resting period. The pretreatment of A549 cells with β-glucan, however, significantly increased the IL-8 and IL-6 production upon a stimulation by *S. aureus* with MOI 60:1 or MOI 100:1.

An analysis of the viability of A549 cells demonstrated that there was no increase in the cytotoxicity in cells exposed to *S. aureus* with MOI 60:1. There was an increase, in the cytotoxicity (16 ± 2%) in cells exposed to *S. aureus* with MOI 100:1 determined as described in Material and Methods. However, there was no difference between the number of viable trained and non-trained A549 cells exposed to *S. aureus* with the MOI 100:1. Since we compared the interleukin production between trained and non-trained cells exposed to *S. aureus* with the same MOI, it suggests that the observed increase in interleukin production relates to the immune training with the inducer.

Altogether, these results demonstrated that the development of innate immune memory occurs in diverse non-immune cells.

### Training of MG-63 and A549 cells with MDP and β-glucan alters histone marks responsible for gene transcription

Given that epigenetic changes largely drive innate immune memory, and that histone acetylation is one of the critical epigenetic modifications, we assessed histone 3 acetylation on lysine residue that is linked to an increased gene expression (20). To exam whether β-glucan training of MG-63 and A549 cells mediates changes in H3K27 acetylation the immunocytochemistry method of detection using H3K27ac antibody was employed.

**Figure 5:**
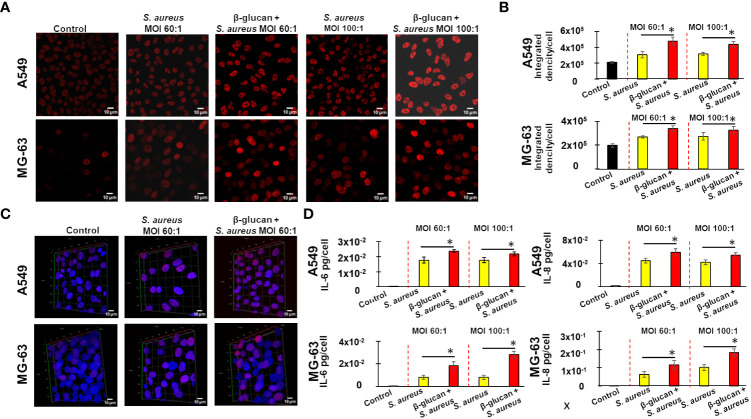
Immunofluorescence confocal microscopy reveals an enhanced H3K27 acetylation in β-glucan-trained cells upon a stimulation by *S. aureus*, with a positive correlation to IL-6 and IL-8 production. Either A549 or MG-63 cells (7,000 cells/well) were seeded in wells of 8wells Labtek culture chambers (Nunc) for 24 h. Afterwards, the cells were either pre-treated with β-glucan, or cultivated in the growth medium for 24 h, followed by a 5-day resting time prior to infection with *S. aureus* for 100 minutes, and then incubated for an additional 24 h as described in Material and Methods. Control cells were cultured in the cultivation medium throughout the experiment. Cells were then fixed with PFA 4%, followed by a permeabilization with Triton X100 0.3% prior to immunocytochemical detection of acetylated histone 3 using primary antibody H3K27ac and secondary Alexa Fluor™ labelled antibody as described in Material and Methods. The experiments were done in a blinded fashion. Five randomly selected fields were used for a quantitative analysis. The representative image of one field is shown. The images of DAPI-stained specimens were captured with strictly identical acquisition settings for every sample using a ZEISS LSM 880 inverted confocal microscope **(A)**. A normalized Integrated Density (ID) of the selected field in the red channel was monitored in order to compare H3K27 acetylation in different samples **(B)**. The vertical red dashed lines differentiate bar graphs corresponding to MOI’s 60:1 and 100:1 of *S. aureus*. Black bar graphs are indicative of control cells. Yellow bar graphs are indicative of cells cultivated in the growth medium for 24 h, followed by a 5-day resting time prior to infection with *S. aureus*. Red bar graphs are indicative of β-glucan-treated cells, which, after a 5 days-resting time, were stimulated by *S. aureus*. Bar graphs **(B)** represent the results from five randomly selected field. To obtain 3D image of cells multiple confocal images acquired at different “z” focal plans from the bottom to the top of the slide culture chamber in the sample. Reconstituted 3D images of host cells were obtained by Z-stack images using a Zeiss ZEN software **(C)**. The representative image of one field is shown. The production of IL-6 and IL-8 in cell supernatants was measured by ELISA **(D)**. The vertical red dashed lines differentiate bar graphs corresponding to MOI’s 60:1 and 100:1 of *S. aureus*. Black bar graphs are indicative of control cells. Yellow bar graphs are indicative of cells cultivated in the growth medium for 24 h, followed by a 5-day resting time prior to infection with *S. aureus*. Red bar graphs are indicative of β-glucan–treated cells, which, after 5 days-resting time, were stimulated by *S. aureus*. Experiments were conducted in triplicate and repeated 3 times. Data are presented as mean +/- SD. The differences among the groups corresponding to different treatments were assessed by analysis of variance (ANOVA) followed by a Tukey’s Honestly Significant Difference test in R software. (*) P-values ≤ 0.05 were considered to be significant.

Microscopy observations of samples viewed in a red channel showed that an exposure of A549 and MG-63 cells to *S. aureus* bacteria with MOI either 60:1 or 100:1 for 24 h resulted in enhanced staining of H3K27. The signal was more intense when cells were trained with β-glucan prior to *S. aureus* exposure (**Figure 5A**). The comparison of the normalized ID (integrated density) between samples acquired in strictly identical conditions by using a semi-quantitative approach, described in Material and Methods section, confirmed this observation. The normalized ID was increased in non-trained cells, which after a 5 day-resting time were exposed to *S. aureus* compared to control untreated cells (**Figure 5B**). Training of cells with β-glucan upon a stimulation with *S. aureus*, following a 5 day-resting time, further increased H3K27 staining (**Figure 5B**).

To rule out non-specific binding, we used an isotype control rabbit IgG antibody under identical conditions as the primary antibody. An isotype control rabbit antibody were of the same class and immunoglobulin type as the primary antibody H3K27ac but have no specificity for any known epitope (Normal rabbit IgG, Sigma-Aldrich, France). In some wells, only a secondary antibody was applied. As a positive control cells were treated with 10 nM of TSA known for its capacity to increase histone acetylation (38). A weak staining was observed in control untreated MG-63 or A549 cells corresponding to the basal level of acetylation in proliferating cells (**Supplementary Figure 1**). Untreated cells incubated with an isotype control antibody instead of H3K27ac antibody or only with the secondary antibody showed no reactivity. In TSA-treated cells a strong coloration was observed that corresponds to the enhanced histone acetylation.

The observation of the 3D image demonstrated the nuclear staining of acetylated H3K27, perfectly co-localized with the blue fluorescence of nuclear DNA, a staining was rather diffuse within the body of the nucleus. The 3D imaging confirmed enhanced staining of acetylated histone in non-trained cells exposed solely to *S. aureus*, following a 5-day resting time. In β-glucan-trained cells, the staining was even more intense after a 5-day resting time prior to exposure to *S. aureus.* The representative image of one field is shown in **Figure 5C**.

To investigate whether there is a potential correlation between epigenetic changes and alterations in interleukin production, we measured the levels of IL-6 and IL-8 in the supernatants of cells that were used for the microscopic analysis. When non-trained A549 or MG-63 cells were exposed to *S. aureus* bacteria with MOI of either 60:1 or 100:1 after a 5-day resting time, there was an increase in the production of IL-6 or IL-8 (**Figure 5D**). The production of IL-6 and IL-8 was even higher in cells that had been trained with β-glucan and cultivated for 5 days prior to exposure to *S. aureus.* The difference was statistically significant. There was a correlation between a H3K27 acetylation in MG-63 and A549 cells and an IL-6 and IL-8 production in supernatants of those cells (**Figures 5B, D**).

To further confirm the increased histone H3K27 acetylation in β-glucan pre-treated cells followed by the exposure *to S. aureus* with histone H3K27 acetylation in cells exposed only to *S. aureus*, MG-63 cells were analyzed by flow cytometry as described previously (36, 37).

To verify the feasibility of using flow cytometry analysis for the evaluation of the level of histone acetylation MG-63, cells were treated with 10 nM of TSA, the inhibitor of the enzymatic activity of HDACs (38). The level of H3K27 acetylation in TSA-treated cells is considered as a positive control. The employment of the isotype control allows to establish the specificity of anti-H3K27ac antibody.

**Figure 6:**
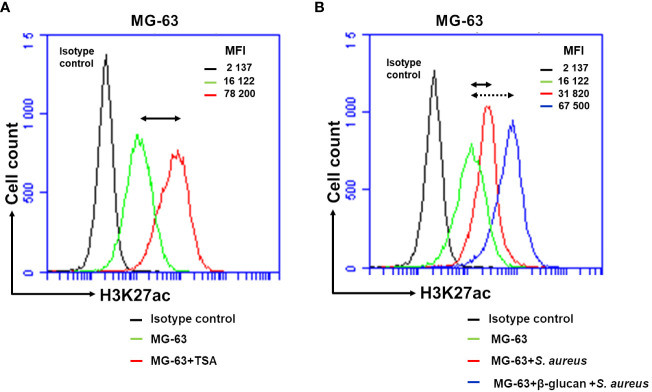
Flow cytometry analysis demonstrates an increased H3K27 acetylation in β-glucan-trained cells upon a stimulation by *S. aureus*. Osteoblasts-like MG-63 cells (20,000 cells/well) were seeded in wells of 24-well plates. Afterwards MG-63 cells were exposed to 100 µg/mL of β-glucan or to the cultivation medium for 24 h, followed by a 5-day incubation prior to infection with *S. aureus* (MOI 60:1) for 100 minutes, and then incubated for an additional 24 h as described in Material and Methods. As a positive control for histone acetylation an incubation of cells with 10 nM of TSA for 24 h were performed. At the end of the incubation, adherent cells were dissociated into single-cell suspension using Trypsin/EDTA treatment. They were then fixed in 4% paraformaldehyde/PBS for 20 min and permeabilized in 0.3% TritonX100/PBS for 10 min. Cells were subsequently incubated with anti-H3K27ac antibody (# 15562, Acetyl-Histone H3 (Lys27) (D5E4) XP^®^ Rabbit mAb, PE Conjugate) diluted in PBS/2%BSA (1:50) for 1 h. An isotype control antibody (#5742, Rabbit (DA1E) mAb IgG XP^®^ Isotype Control, PE Conjugate) was used to exclude nonspecific binding. Afterwards, cells were analyzed for H3K27 acetylation using an Accuri C6 flow cytometer (FL2 channel). Data were collected from 20,000 cells and analyzed using the CFlow software (Becton Dickinson). Values of the respective mean fluorescence intensities (MFIs) were compared to that of the control. Black graph corresponds to the isotype control. **(A)** Double arrow shows the shift between the fluorescence corresponding to the level of acetylation in control cells (represented by the green line) and TSA-treated cells (red line). **(B)** Double arrow shows the shift between the fluorescence corresponding to the level of acetylation in control cells (green line) and in *S. aureus*-infected cells (red line). Dotted double arrow shows the shift between the fluorescence corresponding to the level of acetylation in control cells (green line) and β-glucan pretreated cells exposed to *S. aureus* (blue line). Two independent experiments in triplicate were performed. The results of the one representative experiment is shown.

Basal level of acetylation in control MG-63 cells detected with anti-H3K27ac antibody, corresponding to the level of fluorescence, MFI=16, 122, was higher than the level of fluorescence detected with an isotype control antibody, MFI=2, 137. These results are expected from the presence of acetylated histones in growing cells (**Figure 6A**). A low level of fluorescence detected with the isotype control antibody evidenced the specificity of anti-H3K27ac antibody. The level of fluorescence detected in TSA-treated cells was much higher (MFI=78, 200) than one in control cells (MFI=16, 122) (**Figure 6A**). After a 5-day resting time, exposure of non-trained cells to *S. aureus* resulted in an increase in the level of fluorescence. The level of MIF increased from 16,122 in control cells to 31,820 in *S. aureus*-infected cells. The increase in fluorescence (MIF=67,500) was even higher in cells that had been trained with β-glucan and cultivated for 5 days prior to exposure to *S. aureus* (**Figure 6B**).

The results obtained with flow cytometry analysis are consistent with the results obtained with immunofluorescence confocal microscopy.

### Development of the innate immune memory by non-immune MG-63 and A549 cells depends on reactive oxygen species

**Figure 7:**
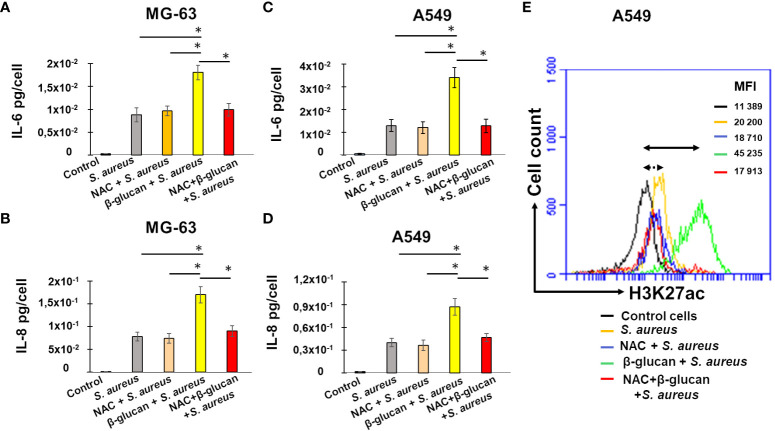
NAC addition before β-glucan-pretreatment decreases IL-6 and IL-8 production upon a stimulation with *S. aureus*. Osteoblast-like MG-63 or lung epithelial A549 cells (20,000 cells/well) were seeded in wells of 24-well plates. Cells were exposed to the cultivation medium or to β-glucan, followed by the incubation for 5 days before an exposure *to S. aureus* at MOIs 60:1, as described in Material and Methods. When indicated cells were pretreated with 1 mM of NAC for 1 h prior to β-glucan and NAC exposure for 24 h or pretreated with NAC for 24 h, followed by the incubation for 5 days before an infection with *S. aureus*. The production of IL-6 and IL-8 was quantified in corresponding supernatants by ELISA and normalized by the number of cells in each well **(A–D)**. Cell supernatants were collected (i) from control untreated cells at the end of experiment (black bar graph), (ii) from cells incubated with a cultivation medium for 24 h, followed by a 5 day-resting period before the exposure to *S. aureus* (grey bar graph), (iii) from cells pretreated with NAC for 1 h and incubated for additional 24 h in the NAC-containing cultivation medium, followed by a 5-day resting period before a *S. aureus* infection (rose bar graph), (iv) from cells pretreated with β-glucan for 24 h, followed by a 5 day-resting period before an exposure to *S. aureus* (yellow bar graph), (v) from NAC-treated cells for 1 h before an exposure to β-glucan and NAC for 24 h, followed by a 5 day-resting period before *S. aureus* infection (red bar graph). The differences among the groups corresponding to different treatments were assessed by analysis of variance (ANOVA) followed by a Tukey’s Honestly Significant Difference test. (*) P-values ≤ 0.05 were considered to be significant. The remaining cells after supernatants collection were used for flow cytometry analysis **(E)** as described in Material and Methods. Briefly, cells were treated with Trypsin/EDTA, and fixed in 4% paraformaldehyde/PBS followed by permeabilization in 0.1% Triton/0.5% BSA/PBS. Cells were subsequently incubated with anti-H3K27ac antibody diluted in PBS/2%BSA (1:50) for 1 h. Cells were then resuspended in PBS and were analyzed for H3K27 acetylation using an Accuri C6 flow cytometer (FL2 channel). Data were collected from 20,000 cells and analyzed using the CFlow software. Cells were analyzed using FSC-A x SSC-A plot. The major density of events was captured by the gate. The events corresponding to debris, cell fragments, and pyknotic cells were eliminated. Values shown on the right side of the graph refer to the respective mean fluorescence intensities (MFIs). The graph shows different lines for various treatments: a black line for untreated control cells, a yellow line for non-trained cells exposed to *S. aureus* (MOI 60:1) after a 5-day resting time, a green line for β-glucan-trained cells exposed to *S. aureus* after a 5-day resting time, a blue line for NAC-treated cells exposed to *S. aureus* after a 5-day resting time, and a red line for NAC-treated cells exposed to β-glucan followed by a 5-day resting time before *S. aureus* infection. Double arrow shows the shift between the fluorescence corresponding to the level of acetylation in control untreated cells (black line) and β-glucan pretreated cells exposed to S. aureus (green line). Dotted double arrow shows the shift between the fluorescence corresponding to the level of acetylation in control untreated cells (black line) and NAC and β-glucan pretreated cells exposed to S. aureus (red line). The results of the one representative flow cytometry analysis is shown.

In order to investigate the mechanisms involved in the development of innate immune memory in non-immune cells to fight *S. aureus* infection, we explored the role of reactive oxygen species by using the ROS scavenger N-Acetyl Cysteine (NAC). In line with the above experiments, exposing MG-63 cells to the medium (non-trained cells), followed by a 5 day-resting time before an exposure to *S. aureus* at MOI 60:1 increased IL-6 and IL-8 production compared to controls. The pretreatment of cells with β-glucan (trained cells) further increased production of IL-6 and IL-8 (**Figures 7A, B**). The addition of NAC to non-trained MG-63 cells, followed by a 5 day-resting time before being exposed to *S. aureus*, did not alter IL-6 or IL-8 production. By contrast, addition of 1 mM of NAC before the β-glucan-pretreatment of MG-63 cells, followed by a 5 day-resting time before being exposed to *S. aureus*, decreased both IL-6 and IL-8 productions upon an exposure to *S. aureus* (**Figures 7A, B**). Similar results were obtained with A549 cells: an addition of 1 mM of NAC prior to β-glucan-pretreatment of A549 cells decreased both IL-6 and IL-8 production upon an exposure to *S. aureus* (**Figures 7C, D**).

To explore the potential correlation between ROS, interleukin production, and histone acetylation, we utilized flow cytometry to analyze the degree of H3K27 acetylation in the remaining cells after supernatant collection and we compared these results with interleukin production levels in the cell supernatants. To determine the involvement of ROS in the development of innate immunity, we used a ROS inhibitor, NAC.

As previously shown, after a 5-day resting period followed by exposure to *S. aureus*, the level of fluorescence detected in infected cells was significantly higher than that measured in control A549 cells (**Figure 7E**, yellow line vs black line). The increase of fluorescence was even higher in β-glucan trained cells, followed by a 5 day-resting time, before a *S. aureus* infection (green line, 45, 235 vs 11, 389). There was no significant differences in MFI= 20, 200 of A549 exposed to *S. aureus*, and MFI= 18, 710 of NAC-pretreated cells before an exposure to *S. aureus* (**Figure 7E**, yellow vs blue line). By contrast, an addition of 1 NAC before the β-glucan-pretreatment of A549 cells followed by *S. aureus* infection, resulted in a decreased MFI, compared to β-glucan-pretreated cells (**Figure 7E**, red line vs green line).

Thus, using two different approaches, ELISA and flow cytometry, we evidenced the correlation in the significant decrease of interleukins production and H3K27 acetylation in cells pretreated with ROS scavenger, NAC, before an exposure to β-glucan, followed by a 5 day-resting time before *S. aureus* infection. These results suggest that the induction of the innate memory of non-immune cells can be mediated, at least in part, by reactive oxygen species.

### The increased production of IL-6 by *Lactococcus lactis* MG1363-trained A549 and MG-63 cells upon a stimulation with *S. aureus*

**Figure 8:**
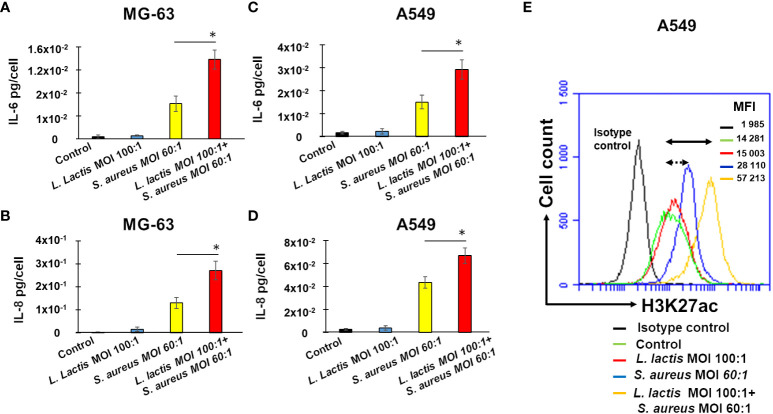
Pre-exposure of MG-63 cells or A549 cells to *L. lactis* increases IL-6 and IL-8 production upon a stimulation with *S. aureus* with a positive correlation to H3K27 acetylation. Osteoblast-like MG-63 cells or lung epithelial A549 cells (20,000 cells/well) were seeded in wells of 24-well plates. Afterwards, cells were exposed either to *L. lactis* or to the cultivation medium for 24 h, followed by the incubation for 5 days before being exposed to *S. aureus* (MOI 60:1), as described in Material and Methods. The production of IL-6 and IL-8 was quantified in corresponding supernatants by ELISA and normalized by the number of cells in each well **(A–D)**. Cell supernatants were collected (i) from control untreated cells at the end of the experiment (black bar graphs), (ii) from cells exposed to *L. lactis* (MOI 100:1), followed by a 5-day resting time and an additional 24 h (bleu bar graphs), (iii) from cells exposed to the cultivation medium for 24 h, followed by a 5-day resting time before an exposure to *S. aureus* (MOI 60:1) (yellow bar graphs), and (iv) from cells exposed to *L. lactis* (MOI 100:1), followed by a 5-day resting time before an exposure to *S. aureus* (MOI 60:1) (red bar graphs. Data are presented as mean +/- SD. The differences among the groups corresponding to different treatments were assessed by analysis of variance (ANOVA), followed by a Tukey’s Honestly Significant Difference test in R software. (*) P-values **≤** 0.05 were considered to be significant. For the evaluation of the level of H3K27 acetylation the remaining A549 cells after supernatants collection were used for flow cytometry analysis **(E)** as described in Material and Methods. Briefly, cells were treated with Trypsin/EDTA, and fixed in 4% paraformaldehyde/PBS followed by permeabilization in 0.1% Triton/0.5% BSA/PBS. Cells were subsequently incubated with anti-H3K27ac antibody diluted in PBS/2%BSA (1:50) for 1 h. An isotype control antibody was used to exclude nonspecific binding. Cells were then resuspended in PBS and were analyzed for H3K27 acetylation using an Accuri C6 flow cytometer (FL2 channel). Data were collected from 20,000 cells and analyzed using the CFlow software. Cells were analyzed using FSC-A x SSC-A plot. The major density of events was captured by the gate. The events corresponding to debris, cell fragments, and pyknotic cells were eliminated. Values shown on the right side of the graph refer to the respective mean fluorescence intensities (MFIs). A black graph corresponds to the isotype control, a green graph corresponds to control untreated cells, a red graph corresponds to cells exposed to *L. lactis*, followed by a 5-day resting time and an incubation for additional 24 h, a bleu graph corresponds to cells exposed to the cultivation medium for 24 h, followed by a 5-day resting time before being exposed to *S. aureus*, an yellow graph corresponds to cells exposed to *L. lactis* followed by a 5-day resting time before being exposed to *S. aureus* at MOI 60:1. Double arrow shows the shift between the fluorescence corresponding to the level of acetylation in control untreated cells (green line) and *L. lactis*-pretreated cells (MOI 100:1) followed by the exposure to *S. aureus* at MOI 60:1 (yellow line). Dotted double arrow shows the shift between the fluorescence corresponding to the level of acetylation in control untreated cells (green line) and *S. aureus*-treated cells (blue line). Two independent experiments in triplicate were performed. The results of the one representative experiments is shown.

In order to analyze the ability of the *L. lactis* reference strain MG1363 to induce innate immune memory against *S. aureus* either in human osteoblast-like MG-63 or lung A549 cells an *in vitro* pilot study was performed. Production of IL-6 and IL-8 by MG-63 cells exposed to *S. aureus* was monitored, with and without preliminary pre-treatment with *L. lactis*, using the method described above to evidence a potential development of innate immune memory. ELISA assessment of IL-6 or IL-8 revealed no differences in IL-6 or IL-8 production between control untreated MG-63 cells and cells exposed to *L. lactis* MG1363 at MOIs 100:1 for 24 h (the training time), followed by a 5 day-resting time and an additional incubation for 24 h (**Figures 8A, B**). An exposure of MG-63 cells to a cultivation medium for 24 h, followed by a 5-day resting time before an exposure to *S. aureus* for 24 h induces a significant increase in IL-6 and IL-8 production, compared to control untreated cells. Pre-exposing cells to *L. lactis* at MOI 100:1 for 24 h (the training period), followed by a resting period of 5 days, and subsequent exposure to *S. aureus* (MOI 60:1), led to a significantly greater increase in IL-6 and IL-8 production compared to cells that were exposed to cultivation medium instead of *L. lactis*, and then rested for 5 days before being exposed to *S. aureus* (**Figures 8A, B**). Similar results were obtained with A549 cells (**Figures 8C, D**).

Furthermore, to investigate the potential correlation between epigenetic alterations and cytokine production, the acetylation of H3K27 in A549 cells was evaluated using a flow cytometry.

The employment of the isotype control (Rabbit (DA1E) mAb IgG XP^®^ Isotype Control) revealed the low level of fluorescence, MFI= 1 985. Basal level of acetylation in control A549 cells detected with anti-H3K27ac antibody Acetyl-Histone H3 (Lys27) (D5E4) XP^®^ Rabbit mAb), MFI=14, 281, was higher than the level of fluorescence detected with isotype control, MFI=1, 985 (**Figure 8E**). An exposure of A549 cells to *S. aureus* after a resting period of 5 days resulted in the increase of the level of fluorescence from 14, 281 in control cells to 28, 110 in infected cells. The increase of fluorescence (MIF=57, 213) was higher in *L. lactis*-trained cells before a *S. aureus* infection (**Figure 8E**).

These findings reveal a significant positive correlation between IL-6 and IL-8 production, as measured by ELISA, and H3K27 acetylation, as evaluated by flow cytometry, in the same experimental conditions. These results strongly suggest that *L. lactis* has the potential to induce innate immune memory against *S. aureus* in non-immune cells.

## Discussion

Staphylococcal infections are becoming a growing worldwide concern, especially in view of antibiotic resistance and escape from host defense mechanisms or by antibiotic usage (41). The dramatic effects of *S. aureus* on human health, as well as the difficulty to achieve efficient immunity against this pathogen, reinforce the need to investigate all means to fight it, including the development of innate immune memory targeting *S. aureus*.

The knowledge regarding the role of innate immune memory during *S. aureus* infection is scarce. Nevertheless, one of the landmark reports on trained immunity (22) showed that β-glucan induced immune training increased resistance to *S. aureus*. Moreover, it has been showed that innate immune memory contributes to the localized host defense in recurrent *S. aureus* skin infection (15). It has to be emphasized that these foundational reports on β-glucan-induced innate immune memory against *S. aureus* infection address immune cell training, while they don’t consider non-immune cell training. *S. aureus* is the main causative agent responsible for the chronic osteomyelitis that leads to progressive bone destruction and loss (42). Initially considered as an extracellular pathogen, *S. aureus* has been detected inside of osteoblasts, where it is likely involved in the development of chronic osteomyelitis (43).

The mechanisms responsible for innate immune memory of immune cells have been intensively studied during the past decade (2, 3, 7, 31). Although fundamental knowledge recently acquired in this field, there is an intriguing conundrum based on the apparent discrepancy between the long-term innate immune memory and the short lifespan of immune cells. Consequently, despite lacking certain defense compounds found in professional immune cells, non-immune cells have attracted attention for their potential to develop innate immune memory due to their longer lifespan. In fact, recent investigations have revealed the development mechanism of innate immune memory in certain types of non-immune cells (5). Nevertheless, the development of innate immune memory by non-immune cells is still a relatively under-studied area. In a recent study, we developed a model of osteoblast-like cells solely bearing *S. aureus* during a long-term infection and showed exacerbated immune and inflammatory responses and signatures of metabolic and epigenetic dysregulation in infected cells (16). Given these findings and the fact that non-immune cells have a longer lifespan compared to immune cells, we investigated the possibility of non-immune osteoblast-like MG-63 cells to develop innate immune memory against *S. aureus* infection. We developed an *in vitro* model to study the induction of innate immune memory in non-immune cells. In order to avoid a state of quiescence that induces changes in gene expression programs and metabolism of cells at a high density (44), the initial cell concentration and the percentage of FCS were adjusted to maintain the culture of MG-63 cells without compromising their proliferative potential until the end of the experiment. This model was further validated with another non-immune cells, the A549 cells line that widely used for the study of *S. aureus*-induced lung cells infection (45, 46). Since the production of cytokines was reported to be altered in cells at a quiescent stage (47), our experimental settings allowed studying cytokine production in optimal conditions.

Taking into account the fact that innate immune memory is dependent on epigenetic remodeling and on alternation of cellular metabolism that in turn lead to a prolonged high protective immune response to efficiently react on a second stimulation (3), we investigated cytokines response and epigenetic marks of β-glucan-trained non-immune cells exposed to *S. aureus*. Using this model we demonstrated an increase of IL-6 and IL-8 production in infected cells, which corroborate our previous results of transcriptome profiling of MG-63 cells during long-term *S. aureus* infection (16). The production of IL-8 and IL-6 was increased even to a greater extent in cells, after a training with β-glucan or with MDP, prior to *S. aureus* infection. It is important to note that the initial concentration of cells was 20,000 cells per well, which slightly increased after 24 h of incubation followed by the exposure with the inducer. By the end of the experiment, the cell count had significantly risen to approximately 300,000 cells per well. The findings suggest that at the end of the experiments, the cell population contains only a part of the cells that were originally exposed to inducers. This means that the detected levels of IL-6 and IL-8 production may have been diluted in the cell culture over the course of the incubation period. Therefore, the production of IL-6 and IL-8 measured in our experiments suggests that some of the cells had retained a memory of the initial exposure to the inducers, which is consistent with the concept of innate immune memory.

As far as we know, there is no direct evidence linking interleukins production and epigenetic alterations in the context of innate immune memory response against *S. aureus* infection in non-immune cells.

The core histones that constitute nucleosomes undergo various post-translational modifications (including acetylation, methylation, and phosphorylation), that impact their interactions with DNA. Acetylation of positively charged lysine residues results in its neutralization, leading to the weakness of histone-DNA interaction, which in turn allows the binding of transcriptional factors and promotes transcription (48). Acetylation of lysine at N-terminal position 27 of the histone H3 (H3K27) is usually associated with enhancers and promoters of active genes (22, 2014). We used immunofluorescence microscopy and flow cytometry with specific anti-H3K27ac antibodies to analyze the acetylation of H3K27 in non-immune A549 and MG-63 cells in the context of innate immune memory against *S. aureus*. According to the results of the current study, increased acetylation of H3K27 in *S. aureus*-infected cells suggests the promotion of gene expression. This increase was even higher in β-glucan-treated cells infected with *S. aureus*. To the best of our knowledge, there is currently no direct evidence linking the production of IL-6 and IL-8 to H3K27 acetylation in the context of innate immune memory response, particularly during *S. aureus* infection of non-immune cells. We performed experiments assessing IL-6 and IL-8 production and changes in H3K27 acetylation in the same experiments. Epigenetic modifications revealed by an increased acetylation of H3K27 was concomitant with an upregulation of IL-6 and IL-8 production in β-glucan-trained MG-63 and A549 cells upon a stimulation with *S. aureus* as was shown by microscopy, flow cytometry and ELISA approaches. A significant positive correlation between IL-6 and IL-8 production and H3K27 acetylation in our experimental setting, suggests the development of immune innate memory against *S. aureus* in non-immune cells, but further studies would be needed to fully characterize the mechanism.

As far as we are aware, this is the first study demonstrating the development of innate immune memory in osteoblasts-like MG-63 and in lung epithelial A549 cells during *S. aureus* infection. Since two different types of non-immune cells were used one can suggest that this phenomenon has a general aspect and was not associated to one particular cell line. We hypothesize that other non-immune cell types can develop such innate immune memory. It is worth noting that both MG-63 and A549 are cancer cell lines. While acquiring bone or lung samples from healthy human subjects can be a challenge, further investigation is necessary to confirm these findings. This includes *in vitro* studies using primary culture cells, as well as *in vivo* experiments using animal models.

In response to *S. aureus* infection, host cells synthesize small reactive molecules, such as ROS, with potent cytotoxic properties that eradicate pathogens (49). We have shown that internalized *S. aureus* triggers ROS-mediated DNA damage thus affecting the genomic integrity and/or regulating a gene transcriptional activation (50). It has been shown that ROS regulate major epigenetic processes, DNA methylation and histone acetylation (51). Recent investigations proved the involvement of ROS in the development of innate immune memory: a production of ROS at the site of a sterile injury provides Drosophila with protection towards subsequent infection (52). Using an *in vitro* model it was established that ROS production is a component of innate immune memory for some stimuli, such as the bacillus Calmette-Guérin (BCG) vaccine (31). In the current study we revealed that a pretreatment with a ROS scavenger, NAC, inhibited the ability of β-glucan to trigger enhanced IL-6 and IL-8 production in response to *S. aureus*. Moreover, we evidenced the significant decrease of interleukins production and H3K27 acetylation in cells pretreated with ROS scavenger, NAC, before an exposure to β-glucan followed by *S. aureus* infection. The exact mechanism by which NAC affects H3K27 acetylation is not fully understood, but it is known that ROS play a role in regulating histone acetyltransferases (HATs) and histone deacetylases (HDACs), which are enzymes involved in histone acetylation and deacetylation, respectively (51). Therefore, by reducing ROS levels, NAC may affect the balance between HAT and HDAC activity, leading to reduced H3K27ac levels. Overall, our experiments, using a ROS inhibitor, NAC, demonstrate a significant positive correlation between IL-6 and IL-8 production and H3K27 acetylation that strongly suggest the implication of ROS in the development of innate immune memory against *S. aureus* by non-immune cells.

One of the new insights of the present investigation is that innate immune memory of non-immune cells does not only increase a pro-inflammatory response during *S. aureus* infection but also comprises another augmented reaction of cells, such as a rise in histone 3 acetylation or ROS activation as was shown by the use of NAC, a ROS inhibitor.

*In vivo* experiments have established that certain microbial components could protect mice from infection by a different microorganism (8). Recently, the hypothesis of the development of probiotic-induced innate immune memory was put forward (25). In the current work, we first demonstrated that pre-treatment of non-immune cells with MDP, or with β-glucan, triggers innate immune memory. These being typical bacterial cell wall components, we then sought such an effect with entire bacterial live cells, *Lactococcus lactis* MG1363 strain, which induce the production of cytokines by the host (53). Indeed, pre-treatment of osteoblast-like MG-63 and lung epithelial A549 cells with live *L. lactis* cells, followed by a resting period of 5 days, and by a subsequent exposure to *S. aureus*, resulted in an increased IL-6 and IL-8 production. Furthermore, our findings illustrate a significant positive correlation between IL-6 and IL-8 production and H3K27 acetylation, in *L. lactis*-trained cells suggesting that *L. lactis* may be potential inducer of innate immune system. However, future investigations are necessary to prove this hypothesis and to understand in details the exact mechanisms of this phenomenon *in vitro* and *in vivo*. We don’t exclude the possibility that *in vivo*, multiple induction stimuli or constant low-level induction stimuli may make this effect even greater. Recently it was shown that a treatment of cows with chronic mastitis with viable *L. lactis* DPC3147 cells or heat-killed *L. lactis* DPC3147 cells stimulated a localized immune response (increased IL-8 concentrations in milk) and increased the cure rates in cows with chronic mastitis. (54) The development of innate immune memory we observed here with *L. lactis* MG1363 *in vitro* might account for these positive outcomes.

Altogether, the results obtained with *S. aureus*-infected non-immune cells are in line with those obtained with immune cells (15). Indeed, we observed a rise in a cytokine secretion, an increased H3K27 acetylation, and upregulation of crucial intracellular signaling pathways such as ROS induction in osteoblast-like MG-63 cells as well as in lung epithelial A549 cells.

Our results suggest the possibility of adding *L. lactis*, to the known inducers of innate immune memory, before surgery as a potential option to prevent staphylococcal infection. Nevertheless, more research is required to elucidate the mechanisms of the induction of innate immune memory by non-immune cells, the confirmation of our findings in *in vivo* investigations as well as the development and the standardization of methods in order to study such therapeutic alternatives.

## Conclusion

We demonstrated for the first time memory-like traits in non-immune osteoblast-like MG-63 and lung epithelial A549 cells that are partially dependent on ROS production. The current investigation provides evidence that in addition to their structural functions and the maintenance of tissue homeostasis, non-immune cells are involved in the defense response of infected host by the development of long-lasting immunological memory. These findings may lead to the development of promising therapeutic approach and to improvement of the clinical outcomes of *S. aureus-*caused infections that would be a significant medical advance.

## Data availability statement

The raw data supporting the conclusions of this article will be made available by the authors, without undue reservation.

## Author contributions

Investigation and methodology: EC, SP, ND, YLG, GJ, and NB. Formal analysis and data curation: GJ, EC and NB. Visualization: ND, YLG and NB. Funding acquisition: YLL, ND and NB. Writing - original draft preparation: ÉG, GJ, ND, EC and NB. Writing - review and editing: ÉG, GJ, YLL, DLW and NB. Supervision: NB. Conceptualization: NB. Administration: NB. All authors contributed to the article and approved the submitted version.
