# Supplementary figures and images for: Development of innate immune memory by non-immune cells during Staphylococcus aureus infection depends on reactive oxygen species
Source: Front Immunol. 2023 May 31;14:1138539. doi: 10.3389/fimmu.2023.1138539 (PMC10264681; doi:10.3389/fimmu.2023.1138539)

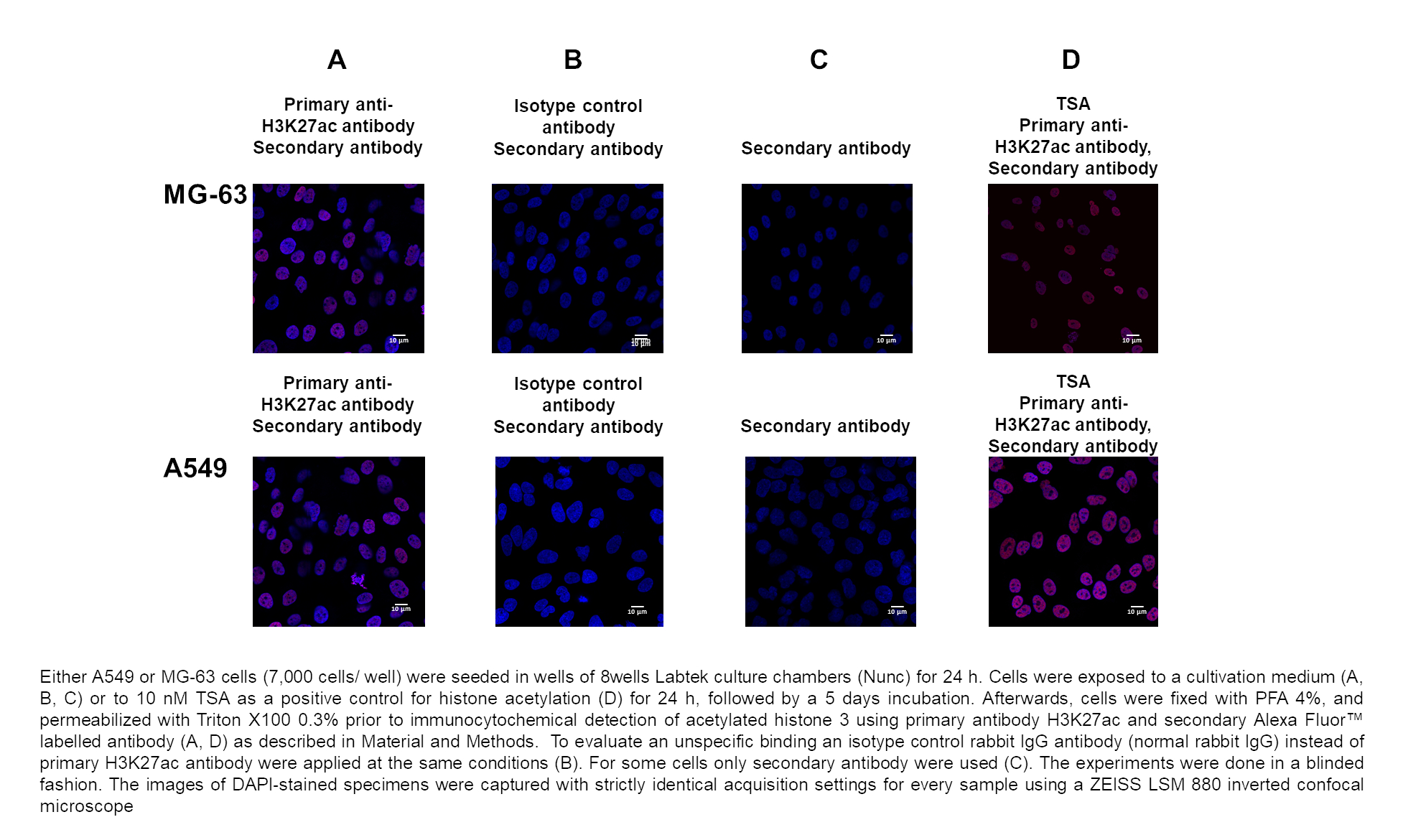

Supplement: Supplementary file 1 [file Image_1.jpg]
